# A Study on the Cutting Characteristics of Bottom Abrasive Grains in Helical Grinding Tools

**DOI:** 10.3390/ma17194814

**Published:** 2024-09-30

**Authors:** Bochuan Chen, Xiaojin Shi, Songmei Yuan

**Affiliations:** 1 School of Mechanical Engineering and Automation, Beihang University, Beijing 100191, China; chenbochuandwx@163.com (B.C.); sxj_zy2307414@buaa.edu.cn (X.S.); 2 Ningbo Institute of Technology, Beihang University, Ningbo 315832, China

**Keywords:** composite ceramics, helical grinding, kinematic analysis, bottom abrasive grain grinding characteristics, pure bottom-edge grains

## Abstract

Helical grinding is crucial for manufacturing small holes in hard-to-machine composite ceramics. This study introduces a geometric model of undeformed chips to analyze the cutting characteristics of abrasive grains on both the bottom and side edges of the tool. It reveals for the first time that the distribution of cutting grains—pure bottom-edge, pure side-edge, and mixed-edge—is influenced by the tool diameter and eccentricity. A novel calculation method for the distribution range (*D_p_*) of pure bottom-edge grains is proposed, demonstrating that using a tool diameter at or below two-thirds of the target hole diameter effectively eliminates pure bottom-edge grains, improving chip evacuation, reducing chip adhesion, and optimizing cutting performance. Experimental validation on small holes in SiC_p_/Al composites (65% volume fraction) confirmed these findings and provides practical guidance for optimizing cutting parameters and tool design.

## 1. Introduction

Composite ceramic materials are ceramics or metal matrix composites containing one or more ceramic particles, fibers, or whisker reinforcement phases [1,2]. These materials are widely used in advanced manufacturing industries. For example, aluminum-based silicon carbide materials (SiC_p_/Al) are utilized in mechanisms for deploying satellite solar panels on space stations, lunar soil drilling equipment, and housings for electronic devices operating under high- and low-temperature conditions [3,4]. SiC fiber-reinforced SiC matrix composites (SiC_f_/SiC) are employed in aircraft engine casings and hot-end turbine blades [5,6]. In the manufacturing of these structures, small hole features are prevalent and challenging to process, posing a significant challenge in the production of composite ceramic components [4,7]. Traditionally, special processing methods, such as electrical discharge machining (EDM) and ultrafast laser machining, are commonly used [8,9]. However, EDM is characterized by low processing efficiency and the presence of a heat-affected layer on the surface, and it can only be used with conductive ceramics [10]. Laser machining, in addition to also producing a heat-affected layer, struggles with effectively controlling the hole taper, especially in high aspect ratio conditions [11,12], resulting in poor exit hole precision and making it difficult to further improve the roundness, cylindricity, and inner wall quality of small holes [13]. Following the completion of these processing methods, it is often necessary to perform additional cutting or grinding operations for finishing, which increases the process flow, reduces processing efficiency, and significantly raises manufacturing costs [14]. The challenge of achieving an efficient and precise machining of small holes in composite ceramic materials remains a bottleneck that limits the large-scale application of these materials.

Using superhard tools like PCD end mills or diamond grinding tools is effective for cutting or grinding small holes in composite ceramics [15,16]. However, PCD tools are expensive, costing several to dozens of times more than diamond grinding tools, and they are less durable [17,18]. In contrast, diamond grinding tools are a more practical option, especially for producing small holes (under 2 mm in diameter) with a length-to-diameter ratio of four or more, offering significant practical value in composite ceramic machining [19]. Diamond grinding tools are classified by manufacturing process into electroplated, brazed, and sintered types [7,20]. Of these, electroplated diamond grinding tools are the most cost-effective, costing just one-tenth of brazed or sintered tools, and they are highly effective for processing small holes in SiC_p_/Al or SiC_f_/SiC.

Due to the single-layer distribution of abrasive grains on electroplated diamond grinding tools, the tools wear quickly [21]. Therefore, a clear understanding of the tool wear mechanism during the machining process is essential to facilitate timely tool replacement and prevent tool failure due to excessive wear, which could damage the workpiece material [22]. Additionally, the large distribution gaps of abrasive grains on electroplated diamond grinding tools can lead to significant axial forces if peck drilling is used for hole-making, causing severe tool wear or even breakage [23]. Thus, a helical grinding process must be adopted to ensure that the tool moves along a helical path, effectively avoiding interference between the grain-deficient areas and the workpiece material [24]. This approach also extends the cutting path through tool rotation at the same layer depth, significantly reducing the undeformed chip thickness and achieving a long tool life. Moreover, the helical grinding process facilitates the entry and exit of lubricant into the cutting zone, significantly enhancing tool life and allowing for direct hole-making on inclined or curved surfaces, offering irreplaceable technical advantages.

The single-layer distribution of abrasive grains on electroplated diamond grinding tools results in rapid tool wear [25]. Therefore, a thorough understanding of the wear mechanisms during machining is crucial for timely tool replacement and preventing tool failure due to excessive wear, which could damage the workpiece. Additionally, the large gaps between abrasive grains can cause high axial force during peck drilling, leading to severe wear or tool breakage [26]. To address these issues, a helical grinding process is recommended. This method ensures that the tool follows a helical path, minimizing interference between grain-deficient areas and the workpiece. It also extends the cutting path through tool rotation at a consistent depth, significantly reducing undeformed chip thickness and enhancing tool longevity [27]. Furthermore, the helical grinding process improves lubricant flow in and out of the cutting zone, substantially increasing tool life and enabling precise hole-making on inclined or curved surfaces, offering distinct technical advantages.

In the helical grinding process, the bottom abrasive grains of the tool remain in continuous contact with the material, leading to poor lubrication conditions and a high tendency for chip adhesion. However, the cutting characteristics of these grains vary significantly depending on their distribution. For example, chip adhesion often occurs at the tool center, where abrasive grain wear is also more severe. While these phenomena are evident, existing research has not clearly explained the wear characteristics of bottom abrasive grains or the variations in wear across different regions of the tool’s bottom edge during helical grinding. This lack of clarity complicates the effective optimization of machining parameters and abrasive tool design, necessitating further research.

Traditionally, the study of material removal mechanisms in helical grinding processes has been based on the kinematic analysis of tools, reflecting changes in the cutting process through variations in undeformed chips [28]. The undeformed chip model proposed by Brinksmeier E [29] in 2008 for helical milling has been widely applied in helical grinding research. Although this model offers a simplified derivation of the side-edge cutting height, it still holds significant reference value. Similarly, Denkena B [30] and colleagues published research on the undeformed chip model for helical milling in 2008, but their model reduces the side-edge cutting height to a linear relationship, lacking critical derivation steps. Both models fail to further elucidate the differences in wear states of bottom abrasive grains based on undeformed chips. Furthermore, other studies in the literature also lack research on the characteristics of bottom abrasive grains in helical grinding processes [27,31].

**Figure 1:**
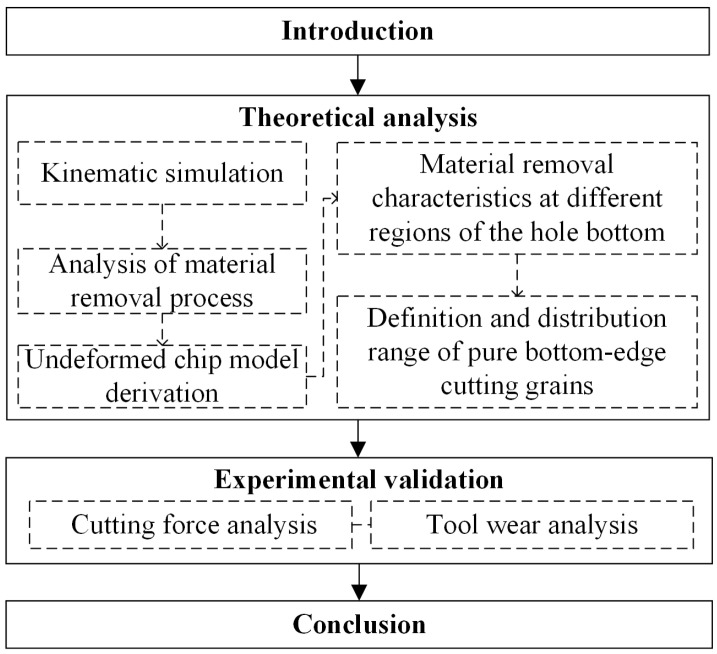
A flowchart of the investigations carried out in this work.

Therefore, to study the cutting characteristics of abrasive grains in different regions of the tool’s bottom edge during the helical grinding of small holes in composite ceramics and to improve tool performance, this paper conducts the following research, which is shown in Figure 1. First, kinematic modeling is performed to establish a total depth model of material removal by the tool’s side or bottom edge during a single revolution at different positions on the hole bottom. Based on this model, the cutting characteristics of abrasive grains at different regions of the tool’s bottom edge are further analyzed. It is found that the tool’s bottom edge simultaneously contains pure bottom-edge cutting grains, pure side-edge cutting grains, and mixed-edge cutting grains involving both edges. The distribution range of these cutting grains is regulated by cutting parameters. This paper defines, for the first time, the distribution range *D_p_* of pure bottom-edge cutting grains. When the tool diameter is 2/3 or less of the target hole diameter, pure bottom-edge cutting grains can be eliminated, significantly reducing chip adhesion on the tool’s bottom edge and optimizing cutting performance. Finally, helical grinding experiments were conducted on small holes in the SiC_p_/Al composite material with a 65% volume fraction. By analyzing cutting forces and observing tool wear, the distribution characteristics and wear patterns of pure bottom-edge cutting grains were verified. By controlling the eccentricity and adjusting the distribution range of pure bottom abrasive grains, tool life can be significantly extended, ensuring the quality of small hole machining.

## 2. Basic Kinematic Parameters

**Figure 2:**
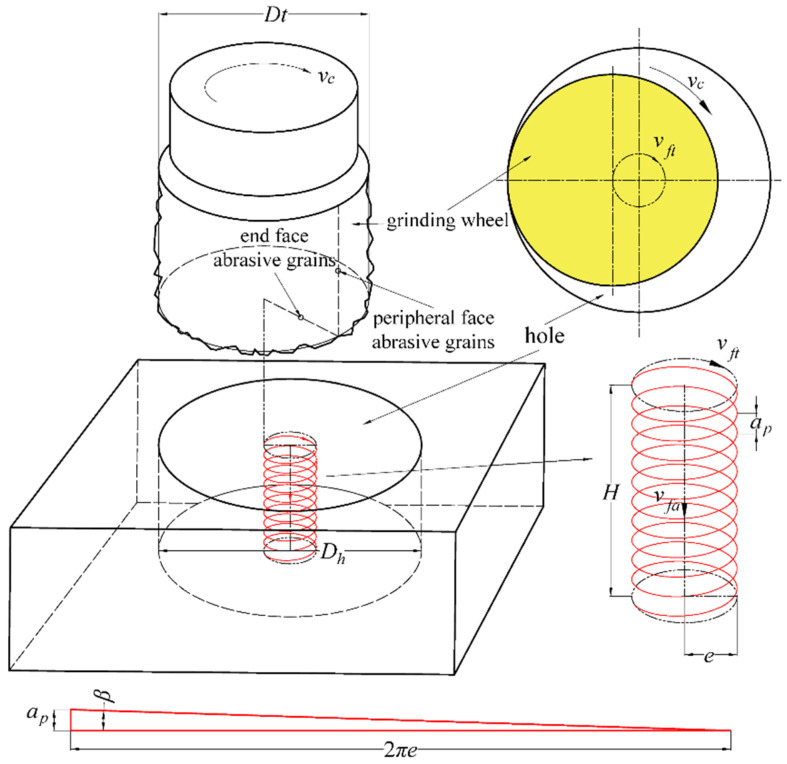
Geometric parameters in helical grinding process.

The main parameters of the helical grinding process are illustrated in Figure 2. For analysis, it is assumed that the grinding wheel is an ideal cylinder that rotates and orbits clockwise in a plane perpendicular to the tool’s axis. During machining, the wheel rotates at a high speed while orbiting at a certain eccentricity. The orbiting speed matches the machine’s horizontal feed speed (*v_ft_*, in mm/min), and its rotational speed aligns with the spindle speed (*n*, in r/min). The cutting speed (*v_c_*, in m/min) is combined with horizontal motion and axial motion (*v_fa_*, in mm/min), which keeps a constant speed, and *v_fa_* typically represents the single-layer cutting depth (*a_p_*, in mm) in the cutting program, indicating the axial advance per orbit. Additional parameters include eccentricity (*e*), the diameter of the hole being machined (*D_h_*), and the diameter of the grinding wheel (*D_t_*) with their geometric relationship, as shown in Equation (1).

(1) $$e=\frac{D_{h}-D_{t}}{2}$$

In helical grinding, the helix angle (*β*) is determined by the relationship between *a_p_* and *e*, as depicted by the displacement triangle shown in Figure 2, where the single-layer cutting depth *a_p_* is equivalent to the pitch. According to the principle that the horizontal helical period equals the axial feed period, the following expression can be found:

(2) $$\frac{v_{fa}}{v_{ft}}=\frac{a_{p}}{2\pi e}$$

(3) $$\beta =\arctan\frac{a_{p}}{2\pi e}=\arctan\frac{v_{fa}}{v_{ft}}$$

## 3. Material Removal Depth Model in Helical Grinding

### 3.1. Kinematic Simulation of Undeformed Chips

**Figure 3:**
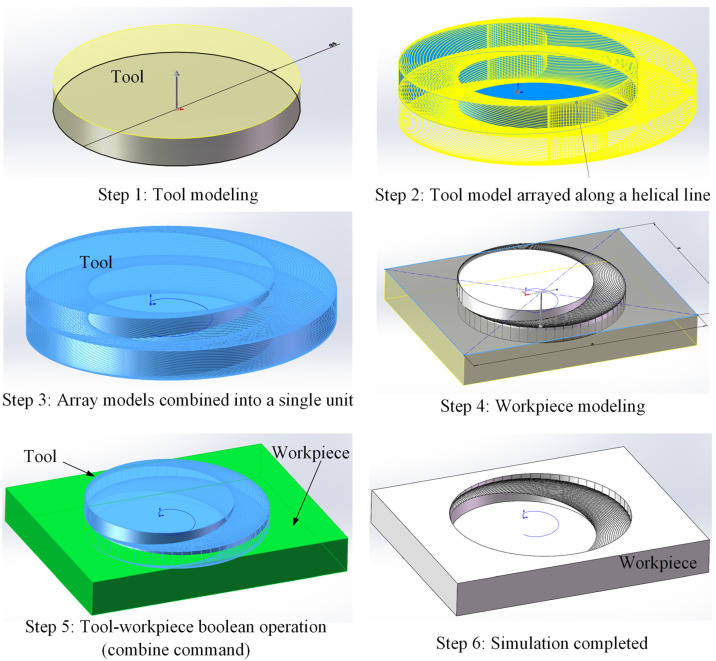
Steps for performing tool kinematic simulation using SolidWorks software (Version: Premium 2020 SP0.0).

To investigate the morphology of undeformed chips from side and bottom abrasive grains during the helical grinding process and determine the cutting removal depth of abrasive grains in different edge regions, kinematic simulations were performed using SolidWorks. The tool’s edge geometry was simplified by assuming that abrasive grains on the bottom edge were uniformly distributed without gaps and that the side edge had no fillets, thus allowing it to be modeled as an ideal cylinder. Additionally, since the simulation is purely kinematic, both the tool and workpiece are treated as rigid bodies, without considering factors such as density, strength, or elastic–plastic deformation. In addition, during the simulation, the tool diameter, the hole diameter, and the pitch of the helical path should be set according to the actual cutting parameters, with the axial feed depth set to the single-layer cutting depth *a_p_*. The simulation process, outlined in Figure 3, involved creating a 3D model of the tool, arraying it along a helical path to form an assembly, and then establishing a geometric model of the workpiece material. A Boolean operation was performed to subtract the overlapping volume between the tool assembly and the workpiece, thus obtaining the morphology of the hole bottom after helical grinding.

**Figure 4:**
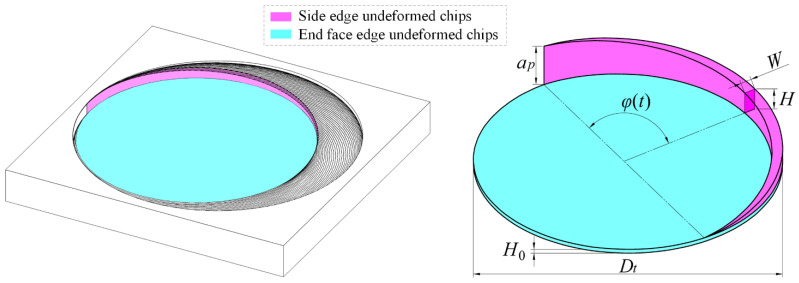
Morphology of undeformed chips in helical grinding.

As shown in Figure 4, the simulation results illustrate a distinct difference in the shapes of undeformed chips generated by the side and bottom edges. By extracting the material removed during one complete rotation of the tool, the undeformed chips for each edge can be obtained. In Figure 4, the undeformed chips from the side edge are depicted as a magenta-shaded crescent-shaped area with thickness *W* and height *H*, both dependent on the instantaneous contact angle *φ*(*t*) of the side edge. The undeformed chips from the bottom edge are shown as a blue-shaded disc-shaped area, with thickness *H*_0_ and a diameter equal to the tool diameter *D_t_*.

### 3.2. Material Removal Process in Helical Grinding

Before determining the relationship between the height of undeformed chips and the contact angle *φ*(*t*), it is crucial to analyze the motion processes of the side and bottom edges. A thorough understanding of the material removal mechanisms in different regions of the hole bottom during the helical grinding process is essential for accurate kinematic modeling.

**Figure 5:**
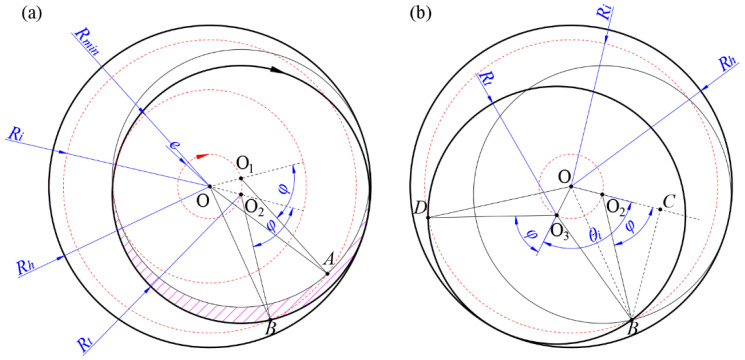
Analysis of helical grinding motion. (**a**) Side-edge cutting process in helical grinding. (**b**) Analysis of bottom-edge cutting depth.

As shown in Figure 5a, both the tool’s rotation and revolution are clockwise. *R_h_* represents the hole radius, *R_t_* is the tool radius, and *R_i_* is the radius of an arbitrary circle centered on the processed hole. O_1_ denotes the initial position of the tool, and O_2_ denotes the position reached after one complete rotation. At this moment, circle *R_i_* intersects circles O_1_ and O_2_ at points A and B, respectively. In Figure 5a, the magenta-shaded area represents the region cut by the tool’s side edge during one complete rotation (the undeformed chip from the side edge). If line O_1_A represents one of the tool’s bottom edges, then O_2_B represents the new position of that bottom edge after one full rotation. By the equality of the three sides in a triangle, it can be demonstrated that $∆{\mathrm{AO}}_{1}O≅∆{\mathrm{BO}}_{2}O$, so $∠{\mathrm{AO}}_{1}O=∠{\mathrm{BO}}_{2}O$, and their supplementary angles are equal, which means that the instantaneous contact angle *φ* of the bottom edge remains constant. Consequently, during one complete revolution of the tool, all projection points of the side edges with an instantaneous contact angle *φ*(*t*) = *φ* are located on a circle with radius *R_i_*.

As the instantaneous contact angle *φ* varies from 0 to π, the trajectory formed by the projection points of the side edge is a series of circles centered at O with radius *R_i_*. When *φ* = 0, *R_i_* is at its maximum radius: *R_i_* = *R_t_* + *e* = *R_h_*; when *φ* = π, *R_i_* is at its minimum radius: *R_i_* = *R*_min_ = *R_t_* − *e*. During one complete revolution of the tool, the area processed by the side abrasive grains is an annular region with a radius between the following values: *R*_min_ ≤ *R_i_* ≤ *R_h_*. Within this region, any point will be ground only once by the side abrasive grains during one full revolution, and the depth of the cut at that time will not fully reach *a_p_*. The bottom abrasive grains will then continue to process that point in subsequent contact periods until the depth of the cut at that point reaches *a_p_*. In the region where *R_i_* ≤ *R*_min_, the side abrasive grains cannot reach, and only the bottom abrasive grains will grind this area. This zone is referred to as the complete bottom-edge removal zone.

From the above analysis, it can be concluded that by determining the height of material removal by the bottom edge at any point on the hole bottom during a single revolution of the tool, one can indirectly deduce the height of material removal by the side edge during the same period. This approach allows for the complete determination of the removal ratio between the bottom and side edges.

### 3.3. Calculation of the Height H_1_ of Undeformed Chips from Bottom Edge

As shown in Figure 5b, during one revolution cycle, when the tool reaches O_2_, point B is cut by the side face abrasive grains for the first and only time, removing a height *H*. As the tool moves between circles O_2_ and O_3_, point B is repeatedly cut by the bottom abrasive grains, removing the remaining height. When the tool reaches circle O_3_, point B is cut by the bottom abrasive grains for the last time, resulting in a cumulative height of *H*_1_. The total height removed by both the side and bottom abrasive grains equals *a_p_*. After this, the tool will no longer contact point B until the next cycle. Therefore, during one complete revolution of the tool, the bottom abrasive grains contribute to the cutting of point B only during the time corresponding to ∠O_2_OO_3_, denoted as *θ_i_*. From the geometric relationship, it can be derived that $∆{\mathrm{BO}}_{2}O≅∆{\mathrm{BO}}_{3}O$, so $∠{\mathrm{BOO}}_{2}=\theta_{i}/2$.

Since the axial feed rate of the tool is constant during helical grinding, the ratio of the central angle corresponding to the feed length in the horizontal plane to the circumferential angle 2π is equal to the ratio of the axial removal depth *H*_1_ to the single-layer cutting depth *a_p_* in the vertical plane. This establishes the following proportional relationship:

(4) $$\frac{\theta_{i}}{2\pi }=\frac{H_{1}}{a_{p}}$$

Therefore, by deriving the expression for *θ_i_*, the specific expression for *H*_1_ can be determined. As shown in Figure 5b, an auxiliary line is drawn from point B to intersect the extended line of OO_2_ at point C. According to the geometric relationships in the figure: OO_2_ = *e*; O_2_B = *D_t_*/2; O_2_C = (*D_t_* cos *φ*)/2; BC = (*D_t_* sin *φ*)/2. In triangle ∆BCO, the following relationship holds:

(5) $$\cos\frac{\theta_{i}}{2}=\frac{OC}{\sqrt{{\left(OO_{2}+O_{2}C\right)}^{2}+BC^{2}}}$$

Substituting the relevant data and rearranging, we obtain the following:

(6) $$\frac{\theta_{i}}{2}=\arccos\frac{e+\frac{D_{t}}{2}\cos\varphi }{\sqrt{\frac{D_{t}^{2}}{4}+eD_{t}\cos\varphi +e^{2}}}$$

By solving Equations (4) and (6) simultaneously, the height *H*_1_ of the material removed by the bottom abrasive grains can be calculated:

(7) $$H_{1}=\frac{a_{p}}{\pi }\arccos\frac{e+\frac{D_{t}}{2}\cos\varphi }{\sqrt{\frac{D_{t}^{2}}{4}+eD_{t}\cos\varphi +e^{2}}}$$

**Figure 6:**
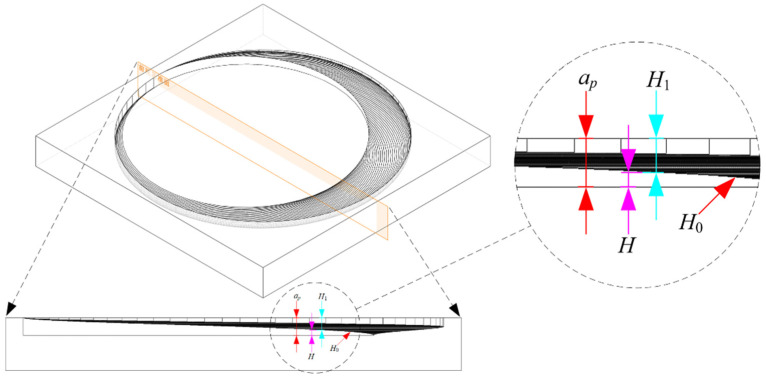
Formation of side-edge cutting depth and relationship between ap/*H*/*H*_1_ and *H*_0._

It is important to note that the removal height *H*_1_ refers to the total height removed by the bottom abrasive grains at any point on the hole bottom during one complete revolution of the tool. *H*_0_ represents the height removed by the bottom abrasive grains during one complete rotation of the tool. As shown in Figure 6, *H*_1_ is the accumulation of *H*_0_. For any point on the hole bottom, the instantaneous *H*_0_ is the same, but the accumulated *H*_1_ varies. The closer the point is to the edge of the hole, the smaller *H*_1_ is. Conversely, the closer the point is to the complete bottom-edge removal zone, the larger *H*_1_ becomes. In the complete bottom-edge removal zone, *H*_1_ is at its maximum and remains constant, equal to the single-layer cutting depth *a_p_*.

### 3.4. Calculation of the Height H of Undeformed Chips from Side Edge

Since any point within the annular region defined by *R*_min_ ≤ *R_i_* ≤ *R_h_* is cut by the side face grains only once during a single revolution cycle, the height *H* of the side-edge undeformed chips can be determined by subtracting *H*_1_ from *a_p_*. The specific steps to solve for the relationship between *H* and *φ*(*t*) are as follows: According to the helical feed principle, after one complete revolution, the axial feed depth is *a_p_*. Thus, any point on the same horizontal plane at the bottom of the hole should descend by *a_p_*. Given that the height removed by the bottom edge is *H*_1_, the following relationship holds for any point at the bottom of the hole:

(8) $$a_{p}=H+H_{1}$$

Substituting Equation (4) into Equation (8), the side-edge cutting depth at point A is as follows:

(9) $$H=a_{p}-\frac{\theta_{i}}{2\pi }a_{p}$$

This means that the cutting depth of the side edge at any point on a circle with radius *R_i_* (corresponding to an instantaneous contact angle *φ*) can be expressed using Equation (9), as shown for point *D* in Figure 5b. By combining Equations (6) and (9), the specific expression for *H* can be derived:

(10) $$H=a_{p}-\frac{a_{p}}{\pi }\arccos\frac{e+\frac{D_{t}}{2}\cos\varphi }{\sqrt{\frac{D_{t}^{2}}{4}+eD_{t}\cos\varphi +e^{2}}}$$

Since there is a one-to-one correspondence between *φ* and *R_i_*, referring to Figure 5b, the following relationship holds in ∆BO_2_O based on the cosine theorem:

(11) $$\cos\left(\pi -\varphi \right)=\frac{{\left(OO_{2}\right)}^{2}+{\left(O_{2}B\right)}^{2}-{\left(OB\right)}^{2}}{2OO_{2}\cdot O_{2}B}$$

Substituting the relevant data and simplifying, the relationship between *φ* and *R_i_* is obtained as follows:

(12) $$\cos\varphi =\frac{R_{i}^{2}-e^{2}-R_{t}^{2}}{2eR_{t}}$$

Substituting Equation (12) into Equation (10) and simplifying yields the relationship between *H* and *R_i_*:

(13) $$H=a_{p}-\frac{a_{p}}{\pi }\arccos\frac{e^{2}+R_{i}^{2}-R_{t}^{2}}{2eR_{i}}$$

In the helical grinding process, the height *H* of side-edge undeformed chips for any point at the bottom can be expressed using either Equation (10) or Equation (13), depending on the application conditions. Equation (13) represents the side-edge undeformed chip model proposed by Brinksmeier E [29] for helical milling. Thus, while the side-edge cutting depth expression derived in this study is consistent with previous models, the derivation method is based on the instantaneous contact angle. This approach provides a more detailed understanding of the material removal process, intuitively reflecting the different removal roles of the side and bottom edges. Additionally, using the instantaneous contact angle as a variable enhances the correlation with the cutting process and facilitates a more effective study of the instantaneous cutting force model.

## 4. Study on the Cutting Characteristics of Abrasive Grains on Tool Bottom Surface

### 4.1. Material Removal Characteristics in Different Regions of the Hole Bottom

**Figure 7:**
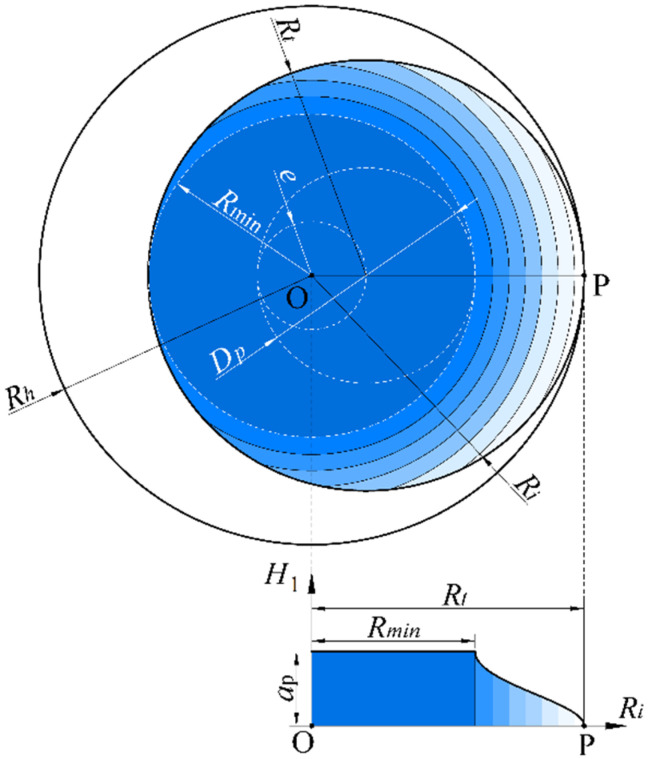
The material removal height *H*_1_ of the bottom edge at different regions on the hole bottom during helical grinding.

As analyzed above, during the helical grinding process, both the bottom edge (bottom abrasive grains) and the side edge (side abrasive grains) contribute to material removal. However, the removal depth of the side and bottom edges varies depending on the position within the hole. When the cutting point is located within the 0-*R*_min_ region, as indicated by the dark blue area in Figure 7, all material is removed by the bottom edge, with no involvement of the side edge. This area is referred to as the complete bottom-edge removal zone, where the height removed by the bottom edge during each revolution is equal to *a_p_*.

When the cutting point is located within the *R*_min_-*R*_h_ region, as shown by the gradient area in Figure 7, the material is removed by both the bottom and side edges. The closer the cutting point is to *R*_min_, the greater the proportion of material removed by the bottom edge; conversely, the closer it is to *R_h_*, the greater the proportion of material removed by the side edge. Therefore, if we take the center of the hole as the origin of the coordinates and expand along any diameter of the hole, the removal depth by the bottom edge can be expressed as a piecewise function:

(14) $$\left\{\begin{array}{l} H_{1}=a_{p}\;(0\leq R_{i}\leq R_{\min})\\ H_{1}=\frac{a_{p}}{\pi }\arccos\frac{e^{2}+R_{i}^{2}-R_{t}^{2}}{2eR_{i}}\;(R_{\min}<R_{i}\leq R_{h}) \end{array}\right.$$

Consider an extreme case: When *e* approaches 0, then *D_t_* = *D_h_*, meaning the tool diameter equals the hole diameter. In this situation, helical grinding essentially becomes conventional drilling, where all material is removed by the bottom edge through pressing and rotating. In contrast, during helical grinding, part of the material is removed by the side edge, which has the highest speed and the best chip evacuation, resulting in the lowest cutting force. Additionally, the rotational motion of the tool significantly increases the travel distance when removing the same volume of material with the bottom edge, thereby greatly reducing the thickness of undeformed chips. The combination of these two factors substantially reduces cutting force during the hole grinding process, highlighting the technical advantage of helical grinding over conventional drilling.

### 4.2. Pure Bottom-Edge Cutting Grains

**Figure 8:**
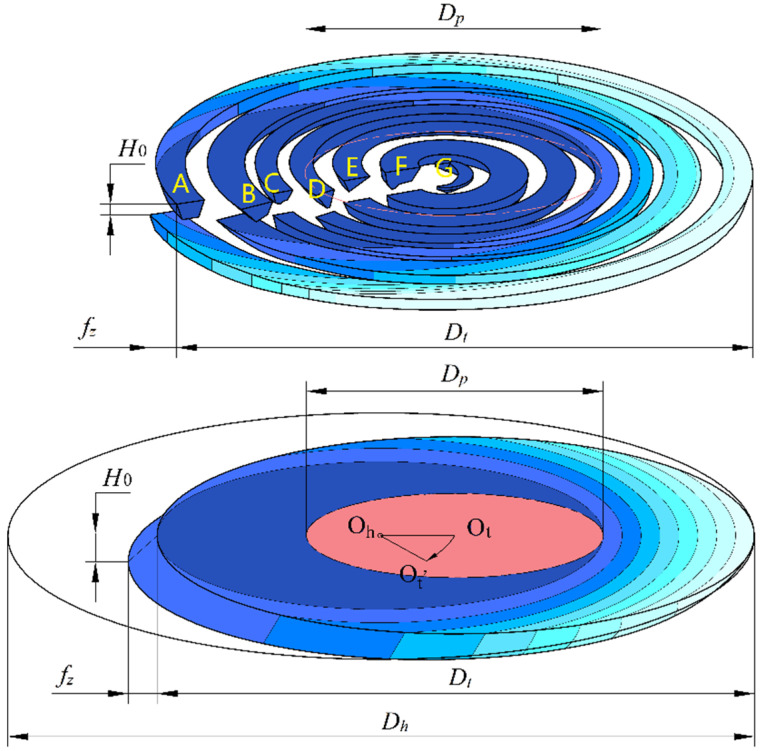
The single rotation removal process of the tool’s bottom edge.

Figure 8 illustrates the material removal process of the bottom edge during a single rotation in helical grinding. The color gradient in the figure represents the variation in removal height by the bottom edge across different areas of the workpiece, with darker colors indicating greater removal heights. O_t_ represents the position of the bottom edge at the start of the tool’s rotation cycle, while O_t’_ represents its position after one full rotation. The arc O_t_ O_t’_ indicates the path length of the tool’s revolution during a single rotation cycle, corresponding to the tool’s feed per revolution. During this process, every effective abrasive grain on the bottom edge participates in the cutting. Imagine the bottom edge of the grinding tool as multiple radial straight edges extending from the center. For any given straight edge, the motion trajectory of the abrasive grains surrounding it follows a three-dimensional helical feed motion, as depicted in the upper half of Figure 8, showing the cutting state. This figure highlights the significant differences in the cutting conditions of abrasive grains at different positions on the bottom edge during a single rotation cycle.

Starting from the tool’s edge, abrasive grain A in the figure can process both the edge of the hole, which is the complete side-edge removal zone, and the area closer to the hole’s center, which is the complete bottom-edge removal zone, within a single rotation cycle. Consequently, the removal depth of abrasive grain A continuously changes during the process, causing variations in the depth it penetrates into the material. This variation allows for the chips formed by grain A to be effectively expelled from the cutting zone.

In contrast, grains near the center of the hole, such as grains E/F/G, experience a constant instantaneous penetration depth at the bottom edge during a single rotation cycle, making it difficult for chips to be expelled from the bottom-edge cutting zone. This can result in chip adhesion and more severe tool wear. Grains between grain A and E, such as grains B/C/D, can cut both the complete bottom-edge removal zone and other areas during a single rotation cycle. Therefore, the instantaneous penetration depth of these grains varies, leading to better chip evacuation performance compared to grains E/F/G.

**Figure 9:**
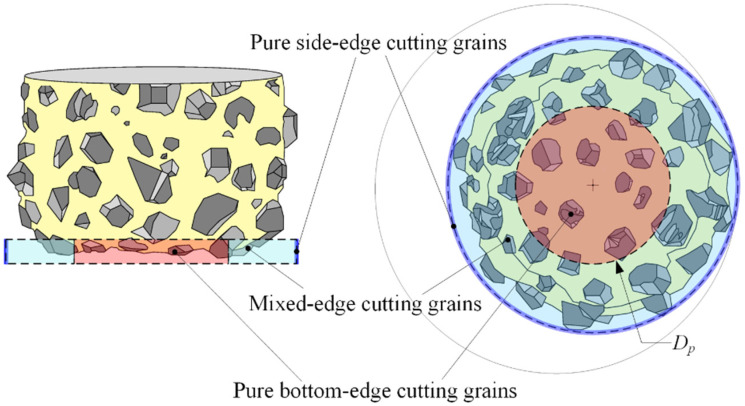
The distribution range of abrasive grains with different removal characteristics on the tool bottom.

Based on the above analysis, it is evident that the cutting conditions of the bottom abrasive grains in the helical grinding process vary significantly. The bottom abrasive grains can be categorized based on their instantaneous penetration depth. As shown in Figure 9, the removal height of grains near the tool’s edge constantly changes, and the outermost ring of grains is referred to as pure side-edge cutting grains. Grains near the tool’s center remain in the pure bottom-edge removal zone and are distributed within a circular area centered on the tool with a diameter *D_p_*. These grains are referred to as pure bottom-edge cutting grains. Grains located between these two regions are classified as mixed-edge cutting grains.

Pure bottom-edge cutting grains can be directly compared to the bottom abrasive grains in conventional hole grinding. They remain in the pure bottom-edge removal zone during the entire process, maintaining a constant instantaneous penetration depth into the material. As a result, their lubrication and chip evacuation conditions are the poorest, making these grains most susceptible to wear and chip adhesion. These grains are the starting point for chip adhesion on the bottom edge and contribute significantly to axial cutting resistance. Therefore, optimizing the performance of these grains is crucial for improving the overall performance of abrasive tools in the helical grinding process.

The derivation of the distribution range *D_p_* for pure bottom-edge grains is as follows: As shown in Figure 7, based on geometric relationships, the radius *R_p_* of the pure bottom-edge cutting grains can be expressed as follows:

(15) $$e=R_{\min}-R_{p}$$

Combining Equation (1) with Equation (15), we obtain the following:

(16) $$D_{p}=3D_{t}-2D_{h}$$

When pure bottom-edge grains do not exist, i.e., when *D_p_* = 0, the following geometric relationship holds:

(17) $$D_{t}=\frac{2}{3}D_{h}$$

## 5. Experimental Validation of Pure Bottom-Edge Abrasive Grain Characteristics

To validate the presence of pure bottom-edge abrasive grains in the helical grinding of small holes, an experimental study was conducted on SiC_p_/Al material. The experiment used a tool of fixed diameter, with the hole diameter controlled by adjusting the eccentricity. This modification changed the distribution range of the bottom-edge abrasive grains. The study investigated the effect of this range on tool performance by observing variations in cutting forces and wear on the bottom edge under different eccentricity conditions.

### 5.1. Experimental Setup

**Figure 10:**
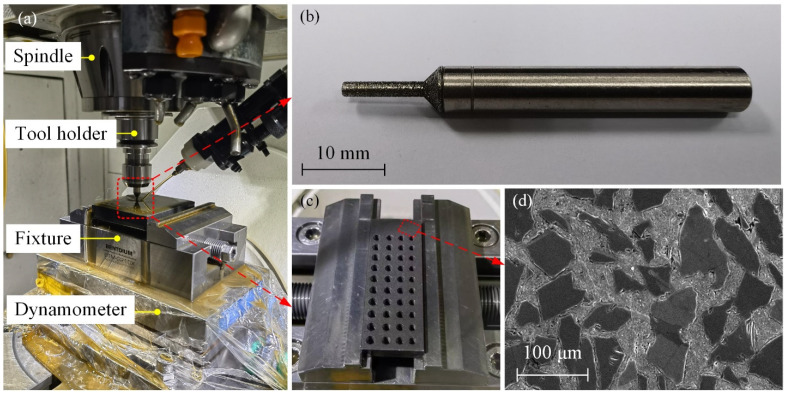
Setup for spiral grinding test on small holes. (**a**) Test site. (**b**) Electroplated diamond abrasive tool. (**c**) Workpiece material. (**d**) Microscopic state of workpiece material.

The experimental setup is shown in Figure 10a. Machining tests were conducted using a Beijing Jingdiao 5-axis machining center (JDGR-200_A10H). Cutting forces during the process were measured with a Kistler 9257B dynamometer (Kistler, Winterthur, Switzerland). The tool used was an electroplated diamond abrasive tool, as depicted in Figure 10b, with a cutting edge diameter *D_t_* of 2 mm, an edge overhang length of 10 mm, a grain size of 100#, and a grain concentration of 100%. The tool was fabricated using electroplating process with a single layer of bonded abrasive grains. During machining, the generated chips can cause reverse abrasion on the tool substrate, resulting in distinct wear marks on the bottom edge. This facilitates the assessment of the distribution range of pure bottom-edge abrasive grains.

The workpiece material used in the experiment was SiC_p_/Al with a 65% volume fraction. The basic parameters of this material are detailed in Table 1, and its macro- and micro-morphologies are shown in Figure 10c and Figure 10d, respectively. This material is a composite metal–ceramic consisting of micron-sized SiC particles as the reinforcement phase and aluminum alloy as the matrix. It is commonly used in electronic packaging applications, such as T/R module packaging and high-power device packaging. These packaging components often feature numerous micro-holes and grooves, which require high machining precision. Due to the high hardness and content of SiC particles, the material exhibits excellent wear resistance, making it highly challenging to machine—a characteristic typical of difficult-to-machine materials.

The cutting parameters were set as listed in Table 2. To ensure effective lubrication and chip evacuation, grinding oil was continuously used for lubrication throughout the process.

Additionally, a new tool was used for each set of parameters. Given that tool wear significantly affects cutting forces, to ensure comparability of the cutting force data, measurements were taken only after the tool had machined 10 holes, allowing it to reach a stable wear state. After machining, the tool was disassembled and subjected to ultrasonic cleaning before observing the wear condition of the tool’s bottom surface. The electron microscope used in the experiment was a Zeiss Gemini 30, and the digital microscope used was a Keyence VHX-6000 (KEYENCE, Itasca, IL, USA).

### 5.2. Data Analysis

Data for an eccentricity of 0.1 mm was excluded from the analysis because this eccentricity is too small relative to the tool radius (less than 10%), making lubrication and chip evacuation extremely challenging and leading to chip clogging. Substituting the relevant parameters into Equation (16) reveals that the pure bottom-edge grinding zone is 1.6 mm, while the gap between the tool and the hole wall is only 0.1 mm. This insufficient gap does not provide an adequate channel for chip evacuation, and it also impedes the entry of the lubricant into the machining area at the bottom edge of the tool. Under these conditions, as the hole depth increases, the bottom edge may cause instantaneous chip adhesion due to ineffective chip removal. Once the material adheres, the bottom abrasive grains are unable to effectively penetrate the material, but the tool continues to feed, leading to an instantaneous overload of the cutting force and tool damage.

**Figure 11:**
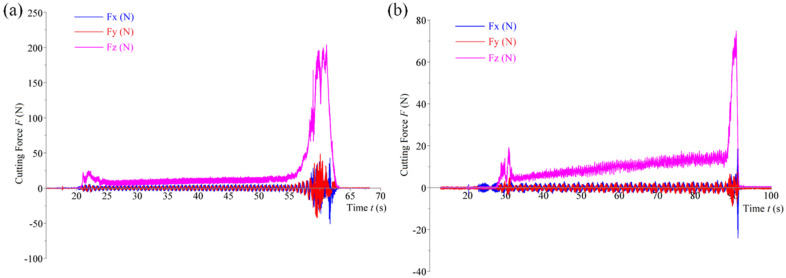
Cutting force at an eccentricity of 0.1 mm. (**a**) Cutting force for tool 1-1. (**b**) Cutting force for tool 1-2.

**Figure 12:**
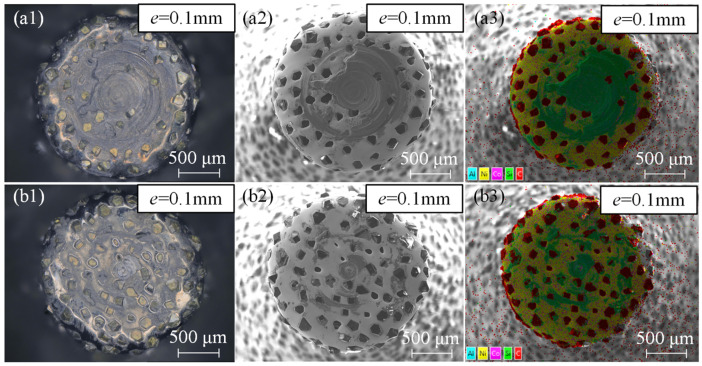
The tool bottom surface wear state at an eccentricity of 0.1 mm in two repeated experiments. (**a1**–**a3**) The optical microscope image, scanning electron microscope (SEM) image, and EDS element distribution map of tool 1-1, respectively. (**b1**–**b3**) The optical microscope image, SEM image, and EDS element distribution map of tool 1-2, respectively.

As shown in Figure 11a,b, the results from two repeated experiments (*e* = 0.1 mm) both indicate that cutting force overload occurred when the cutting depth exceeded 1.6 mm, with the maximum instantaneous cutting force surpassing 200 N. At this point, significant chip adhesion formed on the bottom edge, as depicted in Figure 12a1–b2. The adhered material primarily consisted of Al and SiC, as depicted in Figure 12a3,b3, and the bottom abrasive grains were enveloped by the chips, losing their effectiveness for further machining. Therefore, based on these observations, only experimental data for eccentricities ranging from 0.2 to 0.5 mm were included in the analysis.

**Figure 13:**
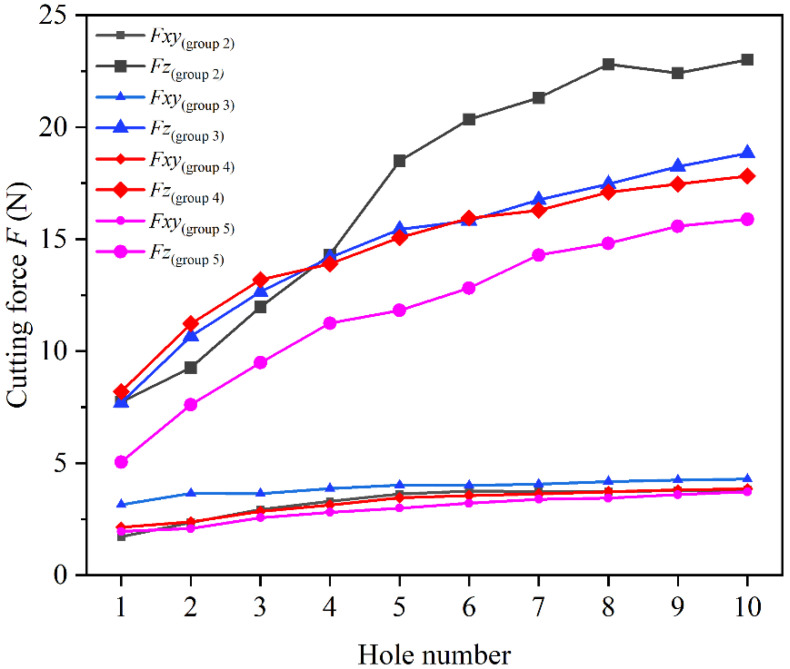
Variations in axial force *F_z_* and radial resultant force *F_xy_* for different holes.

Figure 13 illustrates the variation in cutting forces for four new tools, each processing 10 holes using the parameters from groups 2, 3, 4, and 5 in Table 2. The results indicate that cutting forces increased rapidly during the initial phase and then gradually stabilized. The maximum difference in cutting force between the ninth and tenth holes for each tool was within 2.67%, suggesting that the tools had reached a stable wear state. At this stage, the cutting forces were primarily influenced by the cutting parameters and less by the tool’s wear condition.

### 5.3. Tool Wear Analysis

**Figure 14:**
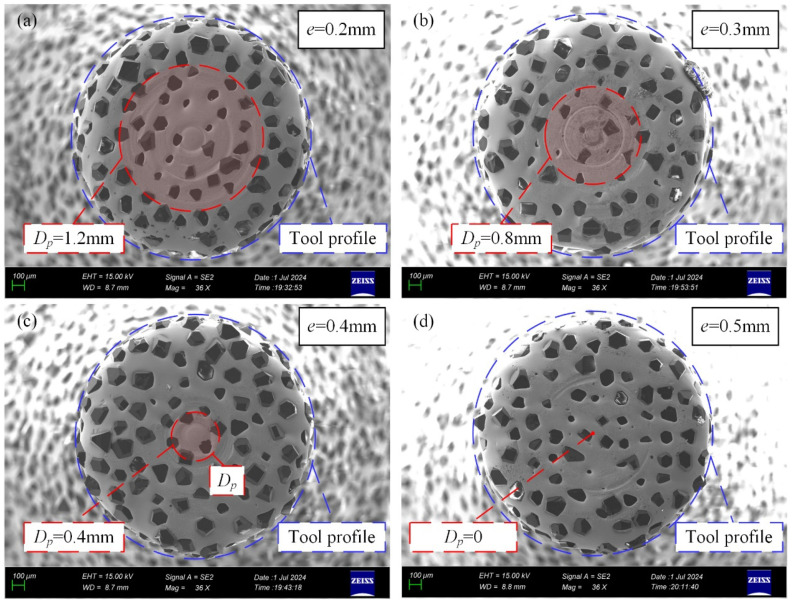
Wear state of 2 mm diameter electroplated diamond abrasive tools and distribution of *D_p_*. (**a**) e = 0.2 mm. (**b**) e = 0.3 mm. (**c**) e = 0.4 mm. (**d**) e = 0.5 mm.

Figure 14 shows the wear condition of the tool’s bottom surface for eccentricities of e = 0.2/0.3/0.4/0.5 mm. In the figure, *D_p_* is represented by red dashed lines and shaded areas. It can be observed that smaller eccentricities lead to a larger distribution range of pure bottom-edge abrasive grains. The extent of the concentric wear marks on the bottom surface closely matches the range of *D_p_*. As shown in Figure 14d, when *D_p_* = 0, the concentric wear marks on the bottom surface nearly disappear, and a uniform wear pattern is observed. These findings indicate that the formation of concentric wear marks is influenced by the size of *D_p_*: the larger the *D_p_*, the greater the distribution range of the concentric wear marks.

**Figure 15:**
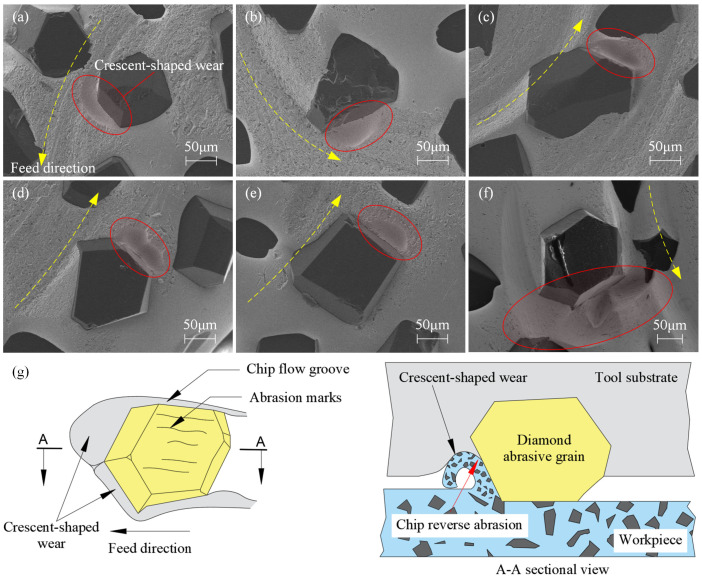
Wear process of pure bottom-edge abrasive grains. (**a**–**f**) Wear marks around pure bottom-edge cutting grains. (**g**) Schematic diagram of wear formation mechanism.

Additionally, upon magnifying the abrasive grains within the *D_p_* range, significant wear marks are evident on the surface and surrounding areas of the pure bottom-edge abrasive grains. As depicted in Figure 15a–f, these wear characteristics include wear marks on the surface of the grains, the chipping of the grains, crescent-shaped wear marks on the rake face of the grains (outlined by the red solid lines), and chip flow grooves on the sides of the grains. The development of these wear features is closely related to the grinding conditions of the pure bottom-edge abrasive grains.

Figure 15g illustrates the wear process of pure bottom-edge abrasive grains. During machining, these grains maintain constant contact with the workpiece material at a fixed depth, resulting in the continuous penetration of their surface into the material. As chips are generated, the limited space for chip evacuation near the grains combined with the higher hardness of the SiC particles relative to the tool substrate causes severe reverse abrasion on the tool substrate. In Figure 15a–f, the yellow dashed lines indicate the tool’s rotation direction. Crescent-shaped wear marks and chip flow grooves develop as chips create sufficient space for evacuation. Subsequently, the chips flow in the opposite direction of the tool’s rotation and exit the cutting zone. As shown by the grains in Figure 15a–f, crescent-shaped wear marks are consistently observed on the rake face of the grains, while uniform chip flow grooves form in the direction of chip flow to facilitate chip removal. These grooves, which counter the tool’s rotational direction, produce numerous concentric wear marks.

Figure 15f illustrates the wear state of pure bottom-edge abrasive grains after machining 50 holes with a single tool. Notable are the larger and deeper crescent-shaped wear marks and more pronounced, extensive chip flow grooves surrounding the grains. This observation suggests that as the tool continues to grind more material and is used for a longer duration, the depth of both the crescent-shaped wear marks and chip flow grooves increases significantly, thereby improving the tool’s chip evacuation performance.

The observed wear conditions indicate that pure bottom-edge cutting grains exhibit the poorest lubrication and chip evacuation capabilities. Therefore, it is essential to minimize the formation of such grains during machining. When selecting a tool for a given hole diameter, it is advisable to choose a tool diameter close to or less than 2/3 of the hole diameter to prevent the occurrence of pure bottom-edge grains. This means that when the tool diameter is 2/3 of the hole diameter, pure bottom-edge cutting grains do not exist. In this case, the penetration depth of all bottom abrasive grains fluctuates, leading to optimal chip evacuation conditions for the bottom edge. Therefore, when selecting a tool based on the hole diameter, it is advisable to choose a tool diameter close to or less than 2/3 of the hole diameter to achieve the best tool life.

Additionally, besides the wear of the abrasive grain surfaces, adaptive crescent-shaped wear marks and chip flow grooves will also develop on the tool substrate during machining. Once these structures stabilize, the tool achieves a steady wear state, optimizing cutting performance.

Since the previous experiments employed different tools, the distribution of abrasive grains on the end face of the tool influenced the variation in cutting forces. To study the impact of the pure bottom-edge grain distribution on cutting forces during helical grinding, the same tool was used to machine micro-holes with varying eccentricities under certain parameters. Similar to the previous tests, conservative parameters were used to process multiple holes before measuring the cutting forces. Once the forces stabilized, indicating that the tool had reached a stable wear state, the main experiment was conducted. Other experimental conditions remained consistent with those in the previous tests. The cutting parameters for this experiment are listed in Table 3.

**Figure 16:**
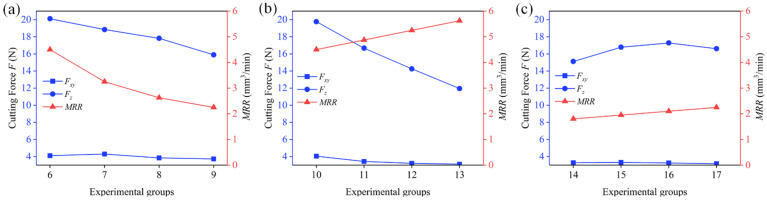
Variations in axial force *F_z_* and radial force *F_xy_* for different test groups. (**a**) Trends of cutting forces and *MRR* with varying *e* when *V_ft_* is constant. (**b**) Trends of cutting forces and *MRR* with varying *e* when *V_fa_* is constant. (**c**) Trends of cutting forces and *MRR* with varying *e* when *β* is constant.

Figure 16 presents the cutting force measurements for machining SiC_p_/Al micro-holes under different parameters using the same tool. Figure 16a shows the results for experiments 6 to 9, where the eccentricity *e* was gradually increased, with constant *v_ft_* and constant *a_p_*. In this case, the feed per revolution *v_fa_* decreased as eccentricity increased, resulting in a gradual reduction in the material removal rate (*MRR*). Figure 16b displays the results for experiments 10 to 13, where eccentricity *e* was increased while multiplying *v_ft_*, keeping *a_p_* constant. Here, *v_fa_* remained constant, leading to a linear increase in the *MRR*. Figure 16c shows experiments 14 to 17, where eccentricity *e* was increased and *a_p_* was proportionally raised, with *v_ft_* held constant. In this case, the helix angle (*β*) remained constant, and the *MRR* increased linearly. The formula used for calculating the *MRR* in this study is as follows:

(18) $$MRR=\frac{a_{p}v_{ft}}{8e}\left(2e+D_{t}\right)$$

As shown in Figure 16a, when the horizontal feed rate remains constant, cutting forces decrease with increasing eccentricity. The maximum reduction in *F_z_* is 21.0%, while *F_xy_* decreases by 9.4% alongside a decrease in the *MRR*. This reduction in cutting force is mainly attributed to the decrease in the axial feed rate resulting from the increased eccentricity despite the horizontal feed rate remaining unchanged. However, this decline in cutting force is influenced by multiple factors, making it difficult to isolate the effect of the pure bottom-edge grain distribution range on the cutting force.

In Figure 16b, when the axial feed rate is kept constant, cutting forces similarly decrease as eccentricity increases. The maximum reduction in *F_z_* reaches 39.6%, and *F_xy_* decreases by 23.2%, while the *MRR* increases significantly. This observation clearly demonstrates that increasing eccentricity, which reduces the pure bottom-edge grain distribution range, not only suppresses cutting forces but also effectively enhances machining efficiency. This validates the method proposed in this paper for reducing the pure bottom-edge grain distribution to improve machining performance.

Furthermore, as shown in Figure 16c, when both horizontal and axial feed rates are held constant and the depth of cut per layer is increased with eccentricity (to maintain a constant helix angle *β*), cutting forces exhibit fluctuations, first increasing and then decreasing. Compared to the 14th group, *F_z_* in the 16th group increased by 9.9%, while *F_xy_* decreased by 3.7%. During the linear increase in the *MRR*, cutting forces exhibited a fluctuation. This phenomenon further supports the conclusion that reducing the pure bottom-edge grain distribution range can effectively suppress cutting forces and improve tool performance.

The results from these variable parameter experiments clearly show that by increasing eccentricity to reduce the pure bottom-edge grain distribution, cutting forces can be significantly reduced even while maintaining an increasing *MRR*.

## 6. Conclusions

Based on theoretical and experimental investigations into the cutting characteristics of abrasive grains in helical grinding processes, the following conclusions can be drawn:

(1). Through derivation and calculation, a geometric model of undeformed cutting chips was developed. Formulas for the cutting heights of the bottom and side edges during tool revolution were derived. The findings indicate that when the cutting point is within the 0-*R*_min_ region, material removal is solely performed by the bottom edge. In the *R*_min_-*R_h_* region, both the bottom and side edges contribute to material removal, with their respective contributions varying according to the specific location.
(2). Abrasive grains on the tool’s bottom edge are categorized into three types: pure side-edge abrasive grains (located at the outermost edge), pure bottom-edge abrasive grains (within the complete bottom-edge removal zone), and mixed-edge cutting grains (intermediate between the other two types).
(3). Experiments involving small hole grinding with a 65% volume fraction of SiC_p_/Al revealed significant concentric wear marks in the areas corresponding to pure bottom-edge grains. These wear marks align with the distribution of these grains and are attributed to insufficient chip evacuation space and the higher hardness of the chips compared to the tool substrate. This results in crescent-shaped wear marks and chip flow grooves.
(4). The results of the variable parameter experiments indicate that increasing the eccentricity, thereby reducing the distribution range of pure bottom-edge abrasive grains, significantly reduces the cutting forces, even when the horizontal feed rate is increased to maintain a constant axial feed rate. This also leads to a substantial increase in the material removal rate (MRR) while reducing the cutting force significantly. These findings demonstrate that the larger the distribution area of pure bottom-edge grains, the greater the cutting forces. Since pure bottom-edge cutting grains exhibit the poorest lubrication and chip evacuation capabilities, it is recommended to select tools with diameters close to or smaller than two-thirds of the hole diameter to mitigate their formation.

The results of this study can guide the selection of cutting parameters for the helical grinding process and provide methods and directions for optimizing tool life. Furthermore, the distribution of pure bottom-edge abrasive grains can serve as an indicator for the design and optimization of abrasive tools. For instance, strategies such as perforating the tool’s bottom surface to reduce the distribution range of pure bottom-edge grains could be investigated. These findings establish a robust research foundation for manufacturing small holes with high length-to-diameter ratios in composite ceramic materials.

## Figures and Tables

**Table 1:** Composition and properties of SiC_p_/Al [32].

Parameter	Value
Average SiC particle size (μm)	80.0
SiC volume fraction (%)	65.0
Thermal conductivity (W/mK, 373.15 K)	73.0
Coefficient of thermal expansion (10^−6^/K)	12.0
Density (g/cm^3^)	3.0
Elastic modulus (GPa)	188.0
Poisson’s ratio	0.3

**Table 2:** Machining parameters under constant helix angle (*β*).

No.	Spindle Speed *n* (rpm)	Eccentricity *e* (mm)	Horizontal Feed Rate *v_ft_* (mm/min)	Single-Layer Cutting Depth *a_p_* (mm)	Axial Feed Rate *v_fa_* (mm/min)	Hole Diameter *D_h_* (mm)	Number of Holes	Hole Depth (mm)
1	30,000	0.1	60	0.05	4.775	2.2	10	8
2		0.2			2.387	2.4		
3		0.3			1.592	2.6		
4		0.4			1.194	2.8		
5		0.5			0.955	3		

**Table 3:** Machining parameters with the same tool.

No.	Spindle Speed *n* (rpm)	Eccentricity *e* (mm)	Horizontal Feed Rate *v_ft_* (mm/min)	Single-Layer Cutting Depth *a_p_* (mm)	Axial Feed Rate *v_fa_* (mm/min)	Hole Diameter *D_h_* (mm)	*MRR* mm^3^/min	*D_p_* (mm)
6	30,000	0.2	60	0.05	2.387	2.4	4.5	1.2
7		0.3	60	0.05	1.592	2.6	3.25	0.8
8		0.4	60	0.05	1.194	2.8	2.625	0.4
9		0.5	60	0.05	0.955	3	2.25	0
10		0.2	60	0.05	2.387	2.4	4.5	1.2
11		0.3	90	0.05	2.387	2.6	4.875	0.8
12		0.4	120	0.05	2.387	2.8	5.25	0.4
13		0.5	150	0.05	2.387	3	5.625	0
14		0.2	60	0.02	0.955	2.4	1.8	1.2
15		0.3	60	0.03	0.955	2.6	1.95	0.8
16		0.4	60	0.04	0.955	2.8	2.1	0.4
17		0.5	60	0.05	0.955	3	2.25	0

## Data Availability

The data presented in this study are available on request from the corresponding author due to ongoing research and further analysis.
